# Implantation and reimplantation of intracranial EEG electrodes in patients considering epilepsy surgery

**DOI:** 10.1002/epi4.12846

**Published:** 2023-11-01

**Authors:** Céline Eelbode, Laurent Spinelli, Marco Corniola, Shahan Momjian, Margitta Seeck, Karl Schaller, Pierre Mégevand

**Affiliations:** ^1^ Neurology division Geneva University Hospitals Geneva Switzerland; ^2^ Clinical Neuroscience Department University of Geneva, Faculty of Medicine Geneva Switzerland; ^3^ Neurosurgery Division Geneva University Hospitals Geneva Switzerland; ^4^ Neurosurgery Division Rennes University Hospital Rennes France; ^5^ INSERM UMR 1099 LTSI, University of Rennes Rennes France

**Keywords:** complications, outcome, seizure onset zone

## Abstract

In patients with drug‐resistant epilepsy who are considering surgery, intracranial EEG (iEEG) helps delineate the putative epileptogenic zone. In a minority of patients, iEEG fails to identify seizure onsets. In such cases, it might be worthwhile to reimplant more iEEG electrodes. The consequences of such a strategy for the patient are unknown. We matched 12 patients in whom the initially implanted iEEG electrodes did not delineate the seizure onset zone precisely enough to offer resective surgery, and in whom additional iEEG electrodes were implanted during the same inpatient stay, to controls who did not undergo reimplantation. Seven cases and eight controls proceeded to resective surgery. No intracranial infection occurred. One control suffered an intracranial hemorrhage. Three cases and two controls suffered from a post‐operative neurological or neuropsychological deficit. We found no difference in post‐operative seizure control between cases and controls. Compared to an ILAE score of 5 (ie, stable seizure frequency in the absence of resective surgery), cases showed significant improvement. Reimplantation of iEEG electrodes can offer the possibility of resective epilepsy surgery to patients in whom the initial iEEG investigation was inconclusive, without compromising on the risk of complications or seizure control.

## 1 INTRODUCTION

In patients with drug‐resistant epilepsy, surgery can bring lasting improvement of seizure control.^1^ When it is not possible to localize the cerebral area generating the seizures with enough certainty using non‐invasive techniques, intracranial EEG (iEEG) might be useful. However, even iEEG fails to localize the region of seizure onsets in 10%–15% of patients.^2^, ^3^, ^4^

In such cases, one may conclude that the patient is not a candidate for curative epilepsy surgery, explant the iEEG electrodes, and consider palliative procedures, like neuromodulation. Indeed, multistaged iEEG implantations are generally discouraged by current international recommendations,^5^ presumably because they are perceived to have an unfavorable balance of risk to benefit for the patient. However, it may also be worthwhile to reconsider the hypotheses that led to the initial implantation scheme, in light of the additional information brought about by the first few days of invasive monitoring. New hypotheses might thus be generated, involving cerebral regions that were not adequately sampled by the initial implantation, and additional iEEG electrodes might be necessary to explore these regions.

Because this second implantation surgery exposes the patient to an additional risk of complications, it is crucial to know whether it is worth it in terms of allowing patients to proceed to epilepsy surgery and with respect to the long‐term control of seizures. Previous case series suggested that revision of iEEG implantation is reasonably safe and effective.^6^, ^7^, ^8^ We conducted a retrospective case–control study to assess the risk of reimplanting iEEG electrodes during the same inpatient stay, and the consequences on long‐term seizure control. Our case–control design allowed us to statistically test whether the risk of complications and the long‐term outcome differed in patients who underwent a second implantation surgery vs those who only had one.

## 2 MATERIALS AND METHODS

### 2.1 Ethical statement

This project was approved by the relevant Institutional Review Body (Commission Cantonale d'Ethique de la Recherche de la République et Canton de Genève; project ID: 2020–01308), which authorized the reuse of clinical data and waived the necessity to obtain written consent from participants, in accordance with Swiss laws.

### 2.2 Cases and controls

We identified 12 patients who underwent implantation and reimplantation of intracranial EEG electrodes at Geneva University Hospitals between 1995 and 2021. We defined reimplantation as a second operative procedure where the position of iEEG electrodes was modified, or additional iEEG electrodes were implanted, in the operative room and under general anesthesia, during the same inpatient stay that started with the initial implantation surgery. The indication for the reimplantation of iEEG electrodes was the failure of the initial implantation scheme to delineate the seizure onset zone, either because the seizures appeared to begin simultaneously in remote cerebral regions or because the onset of clinical features preceded that of the ictal discharge.

We matched each case to one control who had undergone implantation but no reimplantation of iEEG electrodes, out of a list of about 130 patients during the same time span. We used three criteria for matching: duration of epilepsy, temporal lobe vs extra‐temporal lobe epilepsy, and presence or absence of a putative epileptogenic lesion on brain MRI. We selected these three criteria because they are among the main predictors of the success of epilepsy surgery.^9^ We took care to imperatively satisfy the MRI (a single putative epileptogenic lesion vs not a single putative epileptogenic lesion) and epilepsy type (temporal vs extratemporal) criteria first, and then minimized the disease duration criterion. In order to optimize the quality of the pairing, we decided against matching each case to more than one control.

### 2.3 Variables of interest

We counted the number of electrodes and individual contacts implanted in each implantation surgery. An 8 × 8‐contact subdural grid was counted as 1 electrode and 64 contacts. We looked for the following post‐operative complications during the inpatient stay and at the 3‐month follow‐up visit: hemorrhage, intracranial infection, wound infection, and new neurological deficit (except for amputations of the visual field, which were deemed complications of the resective surgery rather than iEEG electrode implantation). We quantified post‐operative seizure control at the last follow‐up (mean duration 32 months, range 3 to 96) using the ILAE classification.^10^

We also examined the results of non‐invasive investigations (focal vs multifocal seizures, interictal EEG, ictal EEG, positron emission tomography, and single photon emission computed tomography).

### 2.4 Statistical analyses

We analyzed binary variables using the McNemar test for correlated proportions,^11^ with correction for continuity,^12^ and continuous variables using Student's paired *t*‐test. We considered seizure control both as a continuous variable and as a binary variable by dichotomizing good (ILAE score 1–3) vs bad outcomes (ILAE score 4–6). We attributed a putative ILAE score of 5 to patients who did not undergo resective surgery, reflecting the absence of any significant change in those patients' seizure frequency and severity. Similarly, we compared the results of non‐invasive investigations between cases and controls both by considering them as continuous variables (attributing scores of 0, 1, and 2 for a normal, monofocal, and multifocal test result, respectively) and as binary variables (normal vs abnormal). We implemented statistical tests in MATLAB (version R2018b, The Mathworks Inc.).

## 3 RESULTS

We matched cases to controls based on the duration of their epilepsy duration, temporal vs extra‐temporal lobe epilepsy, and presence or absence of an epileptogenic lesion on MRI (see Table S1), three factors that are among the most important in predicting post‐surgical seizure freedom.^9^ These patients had long‐lasting (on average 14.5 years elapsed between onset and implantation) and complex epilepsies, MRI‐negative for the most part; furthermore, the results of the non‐invasive evaluation (MRI, interictal and ictal EEG, and PET in all patients, and ictal SPECT in most) failed to converge upon a single putative epileptogenic zone (see Table S2). There were no significant differences between the cases and controls with respect to the results of the non‐invasive investigations.

### 3.1 Illustrative example

A 51‐year‐old woman (Case 6) suffered from focal seizures with impaired awareness and orofacial automatisms since age 16. Interictal EEG showed bilateral independent temporal epileptiform abnormalities, more frequent on the left. Ictal EEG showed right temporal onset in 27 seizures and left temporal onset in 8. MRI showed left‐sided hippocampal sclerosis. PET showed left‐sided temporal hypometabolism that extended into the lateral temporal neocortex. Ictal SPECT showed hyperperfusion of the entire left temporal lobe, as well as the left insula. The initial iEEG implantation targeted the medial temporal lobes (amygdala, anterior and posterior hippocampus) and orbital frontal cortex bilaterally. Seizure onsets were left‐sided in 11 seizures, but appeared to simultaneously involve both medial temporal lobes in 8. After the left amygdalar electrode's position was modified to better sample the amygdala, a further six seizures were recorded, all with a clear left amygdalar onset. A tailored resection of the left amygdala and hippocampus and temporal polar cortex was performed. 23 months after surgery, the patient remained completely free from seizures.

### 3.2 iEEG implantation

**FIGURE 1:**
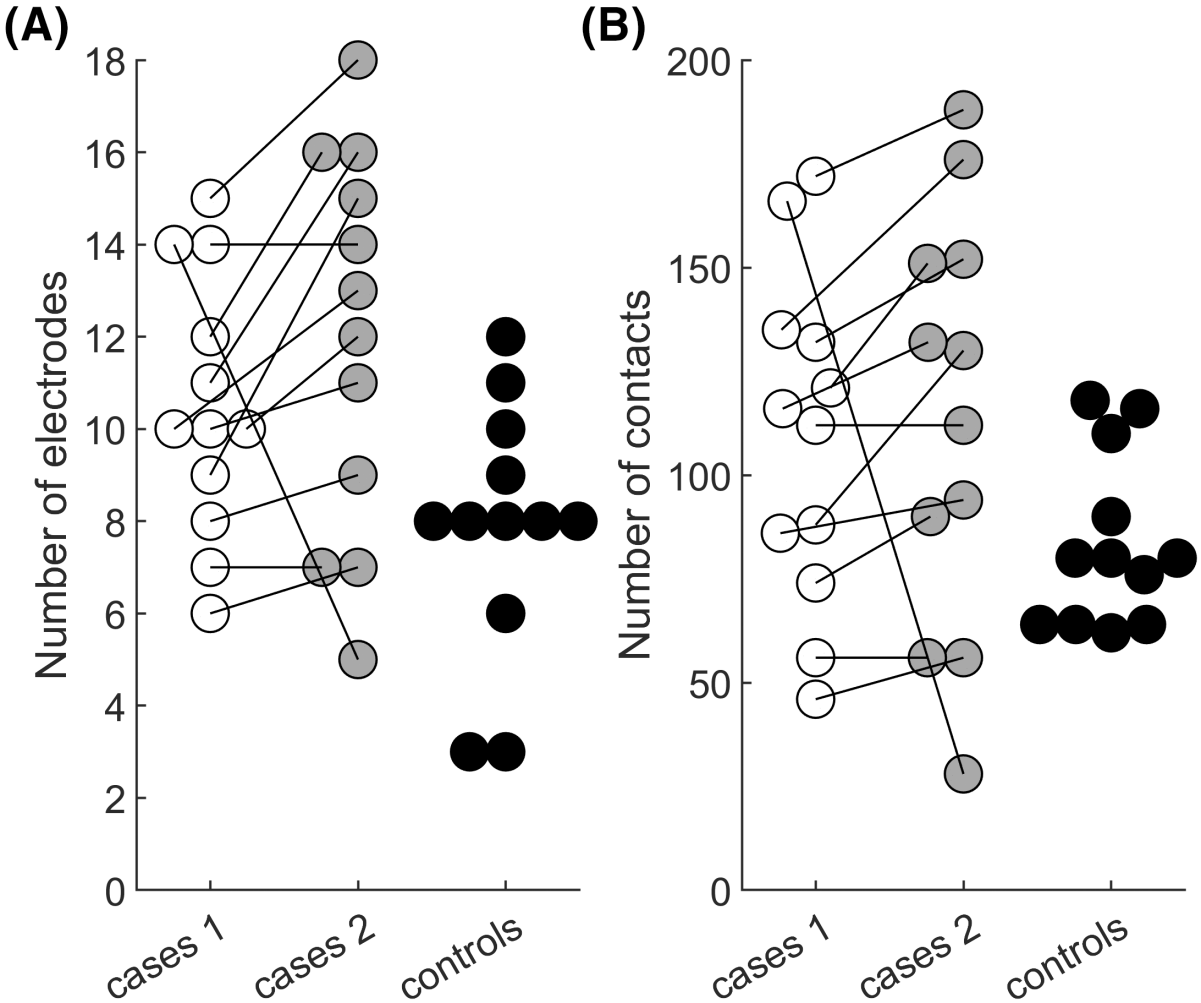
Number of implanted electrodes (A) and individual contacts (B) for the case patients' first and second implantations, and for the controls' implantation. The lines relate each case's first and second implantations.

Figure 1 and Table S3 provide details on the iEEG implantation techniques in each case and control. There were no differences in the number of electrodes and contacts used in the first and the second implantation in case patients (*t*(11) = 1.29, *p* = 0.22; *t*(11) = 0.37, *p* = 0.72, respectively). There was a tendency for more electrodes and contacts to be used in the cases' first implantation vs the controls' (*t*(11) = 2.16, *p* = 0.05; *t*(11) = 2.12, *p* = 0.06). More electrodes were used in the tests' second implantation vs the controls' (*t*(11) = 3.01, *p* = 0.01), and there was a tendency towards more contacts as well (*t*(11) = 1.97, *p* = 0.07).

### 3.3 Complications

None of the case patients suffered from any hemorrhage or infection. One control patient suffered from a post‐implantation epidural and subdural hemorrhage. After undergoing resective surgery, three cases and two controls suffered from a post‐operative neurological deficit. The deficits in the cases were hemispatial neglect in one patient (transient) and verbal memory impairment (permanent) in two. The deficits in controls were an alteration of proprioception (permanent, but slight, and not causing any significant impairment) and trigeminal neuralgia (which appeared soon after, and on the same side as, resective surgery). The proportion of patients who suffered from complications did not differ between cases and controls (*χ* ^2^ = 0, *p* = 1 for all complication types).

### 3.4 Outcome

**FIGURE 2:**
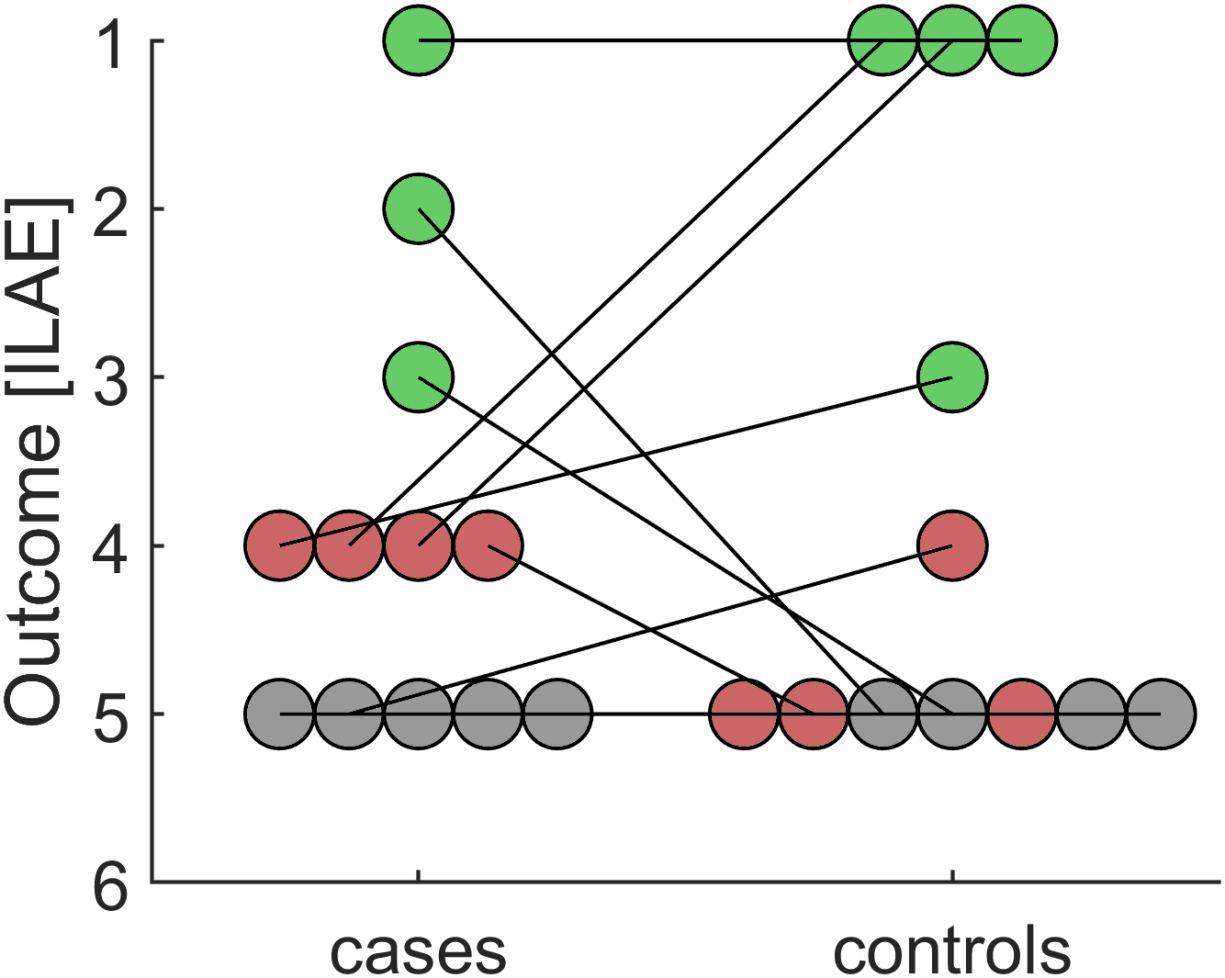
Postoperative seizure outcome. Case–control pairs are linked. Green symbols represent favorable outcome (ILAE score 1–3), red symbols unfavorable outcome (ILAE score 4–6). Gray symbols denote patients who did not undergo resective surgery, and whose seizures did not change significantly in terms of frequency and severity (corresponding to an ILAE score of 5).

Seven cases and eight controls underwent resective surgery (Figure 2 and Table S4). Among the five complete pairs (where both the case and the control patients proceeded to resective surgery), post‐operative seizure control did not differ significantly between cases and controls, whether treated as a continuous variable (mean ILAE score: 3.40 in tests, 2.20 in controls; *t*(4) = 1.50, *p* = 0.21) or as a binary variable (*χ* ^2^ = 1.33, *p* = 0.248). When patients who did not undergo resective surgery were attributed an ILAE score of 5 (reflecting no significant change in seizure frequency and severity), seizure control still did not differ significantly between cases and controls (mean ILAE score: 3.92 in tests, 3.75 in controls; *t*(11) = 0.33, *p* = 0.75; *χ* ^2^ = 0, *p* = 1). Finally, we compared seizure outcomes in reimplanted patients to a hypothetical ILAE score of 5, reflecting their prognosis if no resective surgery could have been offered. Using this approach, we found that reimplantation brought about a significant improvement in seizure control (*t*(11) = −2.86, *p* = 0.02).

## 4 DISCUSSION

In patients with drug‐resistant epilepsy and an inconclusive initial iEEG investigation, our study assessed whether the implantation of additional iEEG electrodes during the same inpatient stay was worth the risk in terms of complications and post‐operative seizure control. We found that reimplantation of iEEG electrodes did not increase complications significantly compared to a single implantation surgery. The majority of reimplanted patients were able to proceed with resective surgery. Seizure control did not differ significantly between cases and controls. Importantly, as a group, patients who underwent reimplantation saw their seizure control improve above what would be expected had the iEEG investigation been declared a failure and no resective surgery attempted. Our findings suggest that reimplantation of iEEG electrodes can offer patients with an inconclusive initial iEEG investigation the possibility of undergoing resective surgery without compromising on either the risk of complications or seizure control.

In our center, the choice of the iEEG implantation technique (stereo‐EEG vs subdural strips and grids) is individualized to each patient's clinical situation. No complication of iEEG electrode implantation occurred in our cases, despite the cumulative risk of two implantation surgeries, and despite the fact that these patients tended to receive more electrodes than controls for each one of their two implantations. Overall, our complication rate is comparable to the numbers reported in the literature.^13^, ^14^

In our series, we did not find that any single examination modality provided more information than the others; rather, the sampling of additional brain regions was guided by a careful reinterpretation of all available evidence in each patient. This underscores the importance of a complete pre‐surgical assessment, including PET and electromagnetic source imaging of interictal activity,^15^ to maximize the patient's chance of benefitting from the iEEG implantation. In cases where the initial iEEG investigation discloses more than one focal seizure onset zone, radiofrequency thermocoagulation through depth electrodes^16^ followed by continued iEEG monitoring might enrich the understanding of the patient's epilepsy and allow definitive treatment of one of the seizure foci.^17^

While the results of epilepsy surgery in our case series may seem modest (average ILAE score below 3, no resective surgery in 5 of 12 patients), they should be weighed against the observation that these patients had long‐lasting and complex epilepsies, MRI‐negative for the most part, and often extra‐temporal, all of which affect the outcome of epilepsy surgery unfavorably.^9^ Furthermore, they faced the perspective of withholding resective surgery completely after the first iEEG implantation failed to identify a focal seizure onset zone.

Three previous case series reported on a total of 48 patients who underwent revision of their initial iEEG electrode implantation during the same inpatient stay^6^, ^7^, ^8^; 38 could proceed to resective surgery. No major complication was reported. Seizure outcome tended to be less favorable than in patients with a single implantation.^6^ Despite our small sample size, our case–control design allowed us to directly compare the risk of complications and the quality of seizure control against patients who did not undergo reimplantation. Our findings extend the literature on revising iEEG implantations and confirm that reimplantation is reasonably safe and offers comparable chances of seizure freedom as in patients in whom the original implantation brought enough information to proceed to surgery.

When planning iEEG implantation schemes, a balance must be sought: on one hand, higher numbers of electrodes increase the risk of complications^18^, ^19^; on the other, insufficient coverage of brain regions could mean that the seizure onset zone is undersampled, or even missed altogether. Importantly, each iEEG electrode samples from a restricted brain region, being most sensitive to generators closer than 1 cm.^20^ Thus, a very focal seizure onset might be missed by an implant that is just slightly misplaced. Clues that the iEEG implantation scheme might have missed the seizure onset zone include EEG seizure onsets that appear simultaneously in remote, but interconnected regions (eg, the hippocampus and cingulate cortex), or that start after the onset of the seizure's clinical features.^7^

While each case should be examined individually, it might prove most beneficial to insert additional electrodes while leaving the originally implanted material in place, rather than explanting it. The targets of these additional electrodes could either densify sampling in a particularly suspect area or explore areas that were considered as potential candidates, but not deemed suspect enough to warrant sampling initially.

To conclude, while we do not advocate for multistaged iEEG procedures as a standard of care, we argue that the safety and efficacy of reimplanting iEEG electrodes are favorable enough to consider this option in patients in whom the first few days of iEEG monitoring have failed to reveal a focal seizure onset zone.

## AUTHOR CONTRIBUTIONS

Conceptualization: PM. Investigation: CE, LS, PM. Visualization: CE, PM. Writing – original draft: CE, PM. Writing—review & editing: MC, SM, MS, KS.

## CONFLICT OF INTEREST STATEMENT

MS is in the advisory board of, and owns shares in, Epilog. The other authors have no conflict of interest to declare.

## ETHICS STATEMENT

We confirm that we have read the Journal's position on issues involved in ethical publication and affirm that this report is consistent with those guidelines.

## Supporting information


Data S1.

## Data Availability

The data that support the findings of this study are available from the corresponding author upon reasonable request.
