# Supplementary material for: Implantation and reimplantation of intracranial EEG electrodes in patients considering epilepsy surgery
Source: Epilepsia Open. 2023 Nov 1;8(4):1622–7. doi: 10.1002/epi4.12846 (PMC10690689; doi:10.1002/epi4.12846)
Supplement: Supplementary file 1 — Data S1. [file EPI4-8-1622-s001.docx]

Supporting information for

**Implantation and reimplantation of intracranial EEG electrodes in patients considering epilepsy surgery**

Céline Eelbode, Laurent Spinelli, Marco Corniola, Shahan Momjian, Margitta Seeck, Karl Schaller, Pierre Mégevand

Geneva University Hospitals, Geneva, Switzerland

**Contents:**

- Supporting table 1. Matching criteria for case-control pairs.
- Supporting table 2. Results of non-invasive evaluation.
- Supporting table 3. Details of iEEG implantations.
- Supporting table 4. Surgeries and seizure outcome.

|  | **Cases** | | | **Controls** | | |
| --- | --- | --- | --- | --- | --- | --- |
|  | **Duration of epilepsy (y)** | **T vs. ET** | **MRI** | **Duration of epilepsy (y)** | **T vs. ET** | **MRI** |
| **1** | 7 | ET (P) | N (stigmata of prior iEEG implantation) | 6 | ET (P) | N |
| **2** | 13 | T | N | 13 | T | N |
| **3** | 13 | T | HS, partial ATLR | 12 | T | HS |
| **4** | 7 | T | N | 13 | T | N |
| **5** | 4 | T | N | 5 | T | N |
| **6** | 35 | T | HS | 40 | T | HS |
| **7** | 7 | ET (P) | N | 9 | O | N |
| **8** | 31 | T | N | 28 | T | N |
| **9** | 8 | ET (C) | N | 8 | F | N |
| **10** | 7 | T | N | 12 | T | N |
| **11** | 15 | ET | N | 13 | ET | N |
| **12** | 20 | ET | N | 21 | ET | N |

Supporting table 1. Matching criteria for case-control pairs. Abbreviations: T, temporal; ET, extratemporal; P, parietal; C, central; O, occipital; F, frontal; N, normal; HS, hippocampal sclerosis; ATLR, anterior temporal lobe resection.

|  | **Cases** | | | | | | **Controls** | | | | | |
| --- | --- | --- | --- | --- | --- | --- | --- | --- | --- | --- | --- | --- |
|  | **Seizure types** | **MRI** | **Interictal EEG** | **Ictal EEG** | **PET** | **SPECT** | **Seizure types** | **MRI** | **Interictal EEG** | **Ictal EEG** | **PET** | **SPECT** |
| **1** | 1 | N | 1 | 1 | 1 | >1 | 1 | N | N | 1 | N | 1 |
| **2** | 1 | N | 1 | 1 | 1 | 1 | 1 | N | 1 | 1 | 1 | ND |
| **3** | 1 | HS, partial ATLR | 1 | 1 | >1 | >1 | 1 | HS | >1 | >1 | 1 | ND |
| **4** | 1 | N | 1 | 1 | 1 | 1 | >1 | N | N | 1 | 1 | >1 |
| **5** | >1 | N | >1 | 1 | N | 1 | 1 | N | >1 | >1 | 1 | ND |
| **6** | 1 | HS | >1 | 1 | >1 | >1 | 1 | HS | >1 | 1 | 1 | N |
| **7** | 1 | N | 1 | 1 | 1 | 1 | 1 | N | >1 | >1 | >1 | >1 |
| **8** | 1 | N | >1 | 1 | >1 | >1 | 1 | N | N | 1 | 1 | 1 |
| **9** | 1 | N | N | 1 | >1 | 1 | 1 | N | 1 | 1 | N | 1 |
| **10** | 1 | N | 1 | 1 | >1 | ND | 1 | N | 1 | 1 | N | >1 |
| **11** | 1 | N | 1 | 1 | N | >1 | >1 | N | >1 | 1 | N | >1 |
| **12** | 1 | N | N | 1 | N | >1 | 1 | N | 1 | 1 | >1 | >1 |

Supporting table 2. Results of non-invasive evaluation. Abbreviations: N, normal; HS, hippocampal sclerosis; ATLR, anterior temporal lobe resection; ND, not done. 1 represents a single seizure type, a single localization of interictal epileptiform discharges on interictal EEG, a single localization of seizure onset on ictal EEG, a single hypometabolic region on PET, and a single hyperperfused region on SPECT; > 1 represents more than one of the abovementioned findings.

|  | **Cases – 1^st^ implant** | | | **Cases – 2^nd^ implant** | | | **Controls** | | |
| --- | --- | --- | --- | --- | --- | --- | --- | --- | --- |
|  | **Electrodes** | **Contacts** | **Technique** | **Electrodes** | **Contacts** | **Technique** | **Electrodes** | **Contacts** | **Technique** |
| **1** | 11 | 132 | SEEG | + 5 | + 20 | + ECoG | 8 | 62 | SEEG, ECoG |
| **2** | 6 | 46 | SEEG | + 1 | + 10 | + SEEG | 8 | 64 | SEEG |
| **3** | 9 | 88 | SEEG | + 6 | + 42 | + ECoG | 8 | 64 | SEEG |
| **4** | 10 | 86 | SEEG | + 1 | + 8 | + SEEG | 11 | 110 | SEEG |
| **5** | 14 | 112 | SEEG | + 0 | + 0 | SEEG | 12 | 118 | SEEG |
| **6** | 7 | 56 | SEEG | + 0 | + 0 | SEEG | 10 | 80 | SEEG |
| **7** | 8 | 116 | ECoG | + 1 | + 16 | + ECoG | 8 | 80 | SEEG, ECoG |
| **8** | 10 | 121 | SEEG | + 3 | + 30 | + SEEG | 8 | 64 | SEEG |
| **9** | 15 | 172 | SEEG | + 3 | + 16 | + ECoG | 9 | 116 | ECoG |
| **10** | 10 | 74 | SEEG | + 2 | + 16 | + SEEG | 6 | 90 | ECoG |
| **11** | 12 | 135 | SEEG | + 4 | + 41 | + SEEG | 3 | 76 | ECoG |
| **12** | 14 | 166 | SEEG | 5 | 28 | ECoG | 3 | 80 | SEEG, ECoG |

Supporting table 3. Details of iEEG implantations. SEEG: stereo-EEG. ECoG: electrocorticography (subdural electrode grids or strips). In cases 5 and 6, existing depth electrodes were repositioned during the second implantation surgery. In case 12, stereo-EEG electrodes were explanted and subdural strip electrodes implanted during the second implantation surgery.

|  | **Surgery** | **Outcome (ILAE)** | **Follow-up duration (months)** |
| --- | --- | --- | --- |
| **T1** | Posterior right cingulate and parietal cortectomy | 4 | 18 |
| **T2** | Temporal left polectomy, left amygdalo-hippocampectomy | 3 | 20 |
| **T3** | Hippocampal medial part resection | 4 | 13 |
| **T4** | Right anterior temporal lobe resection | 2 | 32 |
| **T5** | No resective surgery, DBS implant | NA | 20 |
| **T6** | Temporal left polectomy, left amygdalo-hippocampectomy | 1 | 23 |
| **T7** | Right parietal operculum cortectomy | 4 | 36 |
| **T8** | Temporal right lobectomy with amygdalo-hippocampectomy, insular cortectomy | 4 | 20 |
| **T9** | No resective surgery | NA | NA |
| **T10** | No resective surgery | NA | NA |
| **T11** | No resective surgery | NA | NA |
| **T12** | No resective surgery | NA | NA |
| **C1** | Right medial post-central cortectomy | 1 | 43 |
| **C2** | No resective surgery | NA | NA |
| **C3** | Left anterior temporal lobe resection | 1 | 3 |
| **C4** | No resective surgery | NA | NA |
| **C5** | Right amygdalo-hippocampectomy | 5 | 48 |
| **C6** | Right temporal polectomy, right amygdalo-hippocampectomy | 1 | 47 |
| **C7** | Right occipital craniotomy | 5 | 60 |
| **C8** | Right temporal polectomy, right amygdalo-hippocampectomy | 3 | 48 |
| **C9** | Left frontal cortectomy | 4 | 12 |
| **C10** | Right anterior temporal lobe resection | 5 | 96 |
| **C11** | No resective surgery | NA | NA |
| **C12** | No resective surgery | NA | NA |

Supporting table 4. Surgeries and seizure outcome. T stands for cases, C for controls. Abbreviations: DBS, deep brain stimulation; NA, not applicable.
